# Leukocyte Immunoglobulin-Like Receptors (LILRs) on Human Neutrophils: Modulators of Infection and Immunity

**DOI:** 10.3389/fimmu.2020.00857

**Published:** 2020-05-13

**Authors:** Alexander L. Lewis Marffy, Alex J. McCarthy

**Affiliations:** MRC Centre for Molecular Bacteriology and Infection, Imperial College London, London, United Kingdom

**Keywords:** neutrophil, immune receptor, LILRB2, LILRB3, LILRA2, innate immunity, inhibitory receptor, LILR

## Abstract

Neutrophils have a crucial role in defense against microbes. Immune receptors allow neutrophils to sense their environment, with many receptors functioning to recognize signs of infection and to promote antimicrobial effector functions. However, the neutrophil response must be tightly regulated to prevent excessive inflammation and tissue damage, and regulation is achieved by expression of inhibitory receptors that can raise activation thresholds. The leukocyte immunoglobulin-like receptor (LILR) family contain activating and inhibitory members that can up- or down-regulate immune cell activity. New ligands and functions for LILR continue to emerge. Understanding the role of LILR in neutrophil biology is of general interest as they can activate and suppress antimicrobial responses of neutrophils and because several human pathogens exploit these receptors for immune evasion. This review focuses on the role of LILR in neutrophil biology. We focus on the current knowledge of LILR expression on neutrophils, the known functions of LILR on neutrophils, and how these receptors may contribute to shaping neutrophil responses during infection.

## Introduction

Neutrophils are the most abundant leukocyte in blood circulation and provide the first line of defense against pathogens (1). They sense their environment for signs of microbial infection and rapidly kill invading microbes through three main antimicrobial effector functions: phagocytosis, degranulation, and formation of neutrophil extracellular traps (NETs) (2). Additionally, neutrophils secrete cytokines and inflammatory mediators for recruitment of leukocytes to the infection site (3). Neutrophils are now recognized as complex immune cells that are transcriptionally active (4), signal to other cells, exist as a heterogenous population (5, 6), and contribute to development of inflammatory responses.

Immune receptors have a critical role in neutrophils sensing and responding to their environment. A diverse range of cell surface receptors detect signals of microbial infection, which often induces activation, degranulation, phagocytosis, and NETosis. Signals are often induced through immunoreceptor tyrosine-based activation motif (ITAM) contained within their cytoplasmic tail, or ITAM present on coupled receptors such as the Fc receptor common γ chain (FcRγ) and DAP12. For example, recognition of microbial carbohydrates by C-type lectin (CLEC) receptors can induce neutrophil activation through ITAM-dependent pathways (7–9). Other examples of ITAM-signaling receptors include Fc receptors (FcR) that detect antibody-opsonized microbes (10), carcinoembryonic antigen-related cell adhesion molecule (CEACAM)3 that detects bacterial-derived CEACAM ligands, and sialic acid-binding immunoglobulin-like lectin (Siglec)-14 that detects bacterial-derived Siglec-5 ligands (11, 12). Additional immune receptors function to sense the environment and promote important neutrophil functions such as extravasation, priming, and chemotaxis.

Neutrophil activity is regulated by the expression of inhibitory receptor and paired receptor systems (13). Inhibitory receptors often recognize self-proteins, which induce signaling cascades that raise activation thresholds and suppress immune cell activity. More specifically, inhibitory receptors contain immunoreceptor tyrosine-based inhibitory motifs (ITIMs) in their cytoplasmic tails that become phosphorylated upon ligand recognition. This permits docking of SH2 domain containing tyrosine phosphatases and suppression of downstream signaling cascades. Inhibitory receptors on neutrophils include leukocyte-associated Ig-like receptor 1 (LAIR-1) (14), signal inhibitory receptor on leukocytes 1 (SIRL-1) (15), Siglec-5 (16), leukocyte immunoglobulin-like receptor (LILR)B2 (13), LILRB3 (17), and CEACAM1 (18). ITIM signaling can regulate activation of ITAM signaling receptors, or receptors that couple with ITAM-containing co-receptors including FcRγ or DAP12. Inhibitory receptors are often paired with activating receptor siblings that compete for the same ligands, thereby providing mechanisms to regulate activating signals. Thus, there is compelling evidence that inhibitory receptor, and paired receptor systems, are critical regulators of neutrophil activity, innate immunity, and inflammation.

Here, we review the role of receptors belonging to the LILR multigene family in neutrophil biology. Though there is strong evidence for expression and immunomodulatory functions for individual LILR on leukocytes, many uncertainties remain for their role in neutrophil biology. This is largely because the neutrophil system is difficult to characterize due to their limited transcriptional activity, terminal differentiation and short life. Thus, it is difficult to purify high-quality nucleic acid for transcriptional analysis and it is not possible to genetically manipulate neutrophils for functional analysis. Mouse models have provided insights into neutrophil biology, but observations of paired immunoglobulin-like receptors (PIR; the murine equivalent of human LILR) function often cannot be directly extended to LILR. The problems of studying LILR have been further compounded by a lack of LILR-specific molecular tools. We interrogate evidence of LILR expression on neutrophils from studies utilizing monoclonal antibody staining or mass spectrometry proteomic analysis, and discuss their role in neutrophil biology.

## Leukocyte Immunoglobin-Like Receptors (LILR)

LILR [also known as immunoglobulin-like transcripts (ILTs) or LIR] are a family of 11 receptors that have two to four extracellular Ig-like domains, and are categorized as inhibitory or activating. Most LILR are expressed as membrane-bound receptors, except LILRA3 which is exclusively expressed in a soluble form. The inhibitory receptors (LILRB1 to B5) possess long cytoplasmic tails containing ITIMs, whilst the activating receptors (LILRA1 to A6, excluding A3) possess short cytoplasmic tails and couple with ITAM-bearing FcεRIγ. Individual LILR receptors are classified as group 1 (LILRB1, LILRB2, and LILRA1–3) or group 2 (LILRB3–5 and LILRA4–6) members, based on conservation of LILRB1 residues that recognize human leukocyte antigen (HLA) class I molecules (19). Most *LILR* have limited genetic diversity, except *LILRA6* and *LILRB3* that are polymorphic in their extracellular domains, and *LILRA3* and *LILRA6* that show copy number variation (20–22). Expression of individual LILR has been documented for a range of immune cells including neutrophils, eosinophils, macrophage, dendritic cells, NK cells, B cells, T cells, and osteoclasts and non-immune cells such as endothelial cells and neurons (23). Most *LILR* genes additionally encode soluble forms of LILR produced by alternative splicing (24). *LILR* orthologs found in mice are called PIR; however, there are fundamental differences within human LILR. For example, PIR possess six Ig-like domains and there are only two inhibitory receptors called PIR-B and gp49b1 (25, 26). Human LILRB and PIR-B can modulate the functions of ITAM-bearing receptors such as FcR, B cell receptor (BCR), and T cell receptor (TCR) (27–31). LILR also modulate toll-like receptor (TLR) signaling and functions (32–36). Thus, LILR can modulate a broad set of immune functions, including immune cell function, cytokine release, antibody production, and antigen presentation.

## LILR Expression on Neutrophils

The expression profiles of LILR on neutrophils according to current literature is shown in Table S1. In summary, activating receptors LILRA2, LILRA3, and LILRA5 are expressed on neutrophils. Recent immunoprecipitation and mass spectrometry analysis was unable to confirm the presence of LILRA6 in neutrophil lysates (17). Inhibitory receptors LILRB1, LILRB2, and LILRB3 are expressed on neutrophils, but there is little support for expression of LILRB4 and LILRB5. Further studies are required to characterize expression of LILRA1 and LILRA4.

### Surface-Bound LILR Expressed by Neutrophils

#### LILRA1

LILRA1 (CD86i, LIR6) is a group I receptor that binds to HLA-C free heavy chains but with lower affinities than LILRB1 and LILRB2 (37), and may interact with an unknown *Mycobacterium bovis* ligand (38). LILRA1 is expressed on monocytes and macrophage. Anti-LILRA1 mAb clone m467 does not bind to neutrophils (39). Additionally, all proteomic studies, except one, of neutrophil derived samples have not detected LILRA1-specific peptides. This suggests that LILRA1 is not expressed on neutrophils.

#### LILRA2

Though LILRA2 (ILT1, CD85h, and LIR7) is classed as a group 1 LILR member, it does not interact with HLA-I molecules due to structural differences (40). LILRA2 has been shown to recognize microbially cleaved antibodies (41). LILRA2 expression on neutrophils has been shown using multiple mAb clones (23, 39, 41, 42) and mass spectrometry analyses (43–47). On monocytes, cross-linking of LILRA2 induces calcium mobilization through ITAM signaling of FcRγ (42). It is likely LILRA2 also co-associates with FcRγ on neutrophils. Recognition of truncated antibodies by LILRA2 stimulates ROS production in neutrophils (41). Truncated antibodies are generated by bacterial and fungal proteases suggesting that LILRA2 has evolved to detect microbial infections.

#### LILRA4

LILRA4 (ILT7, CD85g) recognizes bone marrow stromal cell antigen 2 (BST2, also known as tetherin or CD317) (34). LILRA4 is considered to have expression restricted to plasmacytoid dendritic cells (pDCs) and to modulate pDC activity through BST2 (34). There has been no comprehensive assessment of LILRA4-specific mAb binding to neutrophils, and only one study has detected LILRA4 peptides by mass spectrometry analysis of neutrophil samples (45).

#### LILRA5

LILRA5 (ILT11, CD85f, and LIR9) is composed of two extracellular Ig-like domains and remains an orphan receptor. Transcripts of *LILRA5* have been reported in neutrophils (48). More recently, LILRA5-specific peptides have been identified in several proteomic studies of neutrophil derived samples (43–45, 47). However, there remains no comprehensive analysis of LILRA5 expression or cellular location and no assessment of function. Cross-linking of LILRA5 induces monocyte activation and cytokine release (48), suggestive that LILRA5 can stimulate the early phases of immune responses.

#### LILRA6 and LILRB3

The paired receptors LILRB3 (ILT5, CD85a, and LIR3) and LILRA6 (ILT8) possess four homologous Ig-like domains, are polymorphic and display copy number variation (17). LILRB3 is ITIM-bearing, whilst LILRA6 associates with FcRγ. No ligands have been characterized for LILRA6 and LILRB3, though they could interact with a cytokeratin eight-associated ligand on necrotic glandular epithelial cells (49). Monoclonal antibodies raised against LILRB3 and LILRA6 are cross-reactive (17). Thus, additional methods have been required to discriminate expression of LILRB3 from LILRA6, such as transcriptomic or proteomic analyses. Proteome analyses detected LILRB3 and LILRA6 expression in neutrophils (43, 45, 50). However, LILRB3 but not LILRA6 was identified in neutrophil lysates by mass spectrometry analysis following immunoprecipitation and analysis for LILRB3- and LILRA6-specific peptides (17). LILRB3 is released from the surface through an uncharacterized mechanism upon degranulation (17). This raises the possibility that LILRB3 modulates the maturation and/or early activation of neutrophils. Cross-linking of LILRB3 using mAb suppresses FcR-mediated degranulation, phagocytosis, and microbial killing, indicating that LILRB3 has inhibitory capacity (17).

#### LILRB1

LILRB1 (ILT2, CD85j, and LIR1) contains four Ig-like domains and is best-characterized as a receptor for classical and non-classical HLA-I molecules. LILRB1 and HLA-I molecule interactions are dependent on the non-polymorphic α3 domain of the heavy chain and β2 microglobulin. LILRB1 additionally interacts with the UL18 molecules from human cytomegalovirus (HCMV) (51), which is an HLA-I homolog, RIFIN molecules from *Plasmodium falciparum* (52), Dengue virus through an unidentified ligand (53), and the damage associated molecular pattern proteins S100A8 and S100A9 (54). Surface expression of LILRB1 is detectable on a broad range of myeloid cells. Early studies reported conflicting evidence for binding of anti-LILRB1 mAb to neutrophils surface. Notably, Tedla et al. (39) documented binding of anti-LILRB1 clone m402 mAb to neutrophils from 80% of donors. This is supported by detection of anti-LILRB1 mAb clone 292305 binding to neutrophils (55). In contrast, anti-LILRB1 clones VMP355, HP-F1, and GHI/75 are reported to not bind to neutrophils (29, 31, 56). Recent proteomic analyses of neutrophils have detected LILRB1-peptides in neutrophil samples (43, 45, 46, 57, 58). These data suggest LILRB1 is expressed on neutrophils and that epitopes to certain anti-LILRB1 mAb are not accessible or are absent, potentially through alternative splicing or glycosylation patterns.

#### LILRB2

LILRB2 (ILT4, CD85d, and LIR2) contains four Ig-like domains and is a receptor for classical and non-classical HLA-I molecules (19, 59–61). Further ligands characterized for LILRB2 include the class-I like proteins CD1c and CD1d, angiopoietin-like proteins, Nogo receptor ligands, complement split products (CSPs) and β-amyloid (62–66). Several independent studies have documented low levels of LILRB2 expression at the neutrophil surface using multiple mAb clones (23, 31, 39, 55, 67). In addition, several proteomic analyses support the conclusion that LILRB2 is expressed on neutrophils (43–47, 50, 58, 68, 69). Baudhuin et al. (31) localized expression of LILRB2 to granules, and demonstrated that neutrophil stimulation with fMLP, LPS or TNFα lead to rapid surface upregulation which was concomitant with granule exocytosis. Therefore, LILRB2 modulates immune responses during mid- and late-activation phases of the neutrophil lifecycle. Cross-linking of LILRB2 with HLA-G suppresses degranulation and phagocytosis by neutrophils (31). Future studies are required to shed light on how neutrophil and immune responses are modulated through LILRB2 interactions with this diverse set of ligands.

#### LILRB4

LILRB4 (ILT3, CD85k, and LIR5) contains two Ig-like domains and is characterized as a receptor for apolipoprotein E (ApoE) and as a modulator of tolerization of antigen-presenting cells (70–72). LILRB4-specific mAb clone m451 did not bind to the surface of neutrophils (39). In agreement, all but one proteomic analyses of neutrophil samples have not detected LILRB4-specific peptides (45). Bankey et al., documented low and highly variable LILRB4 expression on neutrophils; however, their study used anti-LILRB4 pAb for detection that has cross-reactivity with LILRB1, LILRB2, and LILRB3 (55). Collectively, the data indicates that LILRB4 expression is absent or below detection limits in human neutrophils.

#### LILRB5

Inhibitory receptor LILRB5 (LIR-8, CD85C) possesses four Ig-like domains and ITIMs within the cytoplasmic tail. LILRB5 is an orphan receptor but may interact with an unknown *M. bovis* ligand (38). Expression of LILRB5 has remained relatively unclear, though LILRB5 expression was recently reported on T cells and at lower expression levels on monocytes and dendritic cells (38). Two anti-LILRB5 mAb clones, m481 and ZM3.8, did not bind to neutrophils (39, 70). LILRB5-specific peptides have not been detected in mass spectrometry analysis of neutrophil-derived samples (23). These data suggest that LILRB5 expression is absent or low on neutrophils.

### Soluble LILR Released by Neutrophils

#### LILRA3

LILRA3 (ILT6, CD85e, and LIR4) has four Ig-like domains but no transmembrane or cytoplasmic domains. LILRA3 binds to HLA-I molecules, but with a lower affinity than LILRB1 and LILRB2 (37). Thus, it has been proposed that LILRA3 provides a mechanism to regulate LILRB1- and LILRB2-mediated cellular inhibition through ligand competition. Intracellular stores of soluble LILRA3 have been detected in monocytes and T cells (73). Soluble LILRA3 is released from cells stimulated with IFN-γ and IL-10, and is detected in a variety of bodily fluids including serum (73). In neutrophils, mass spectrometry analysis has repeatedly identified LILRA3 expression (43–46, 50, 58), and has localized LILRA3 to the gelatinase and ficolin granules (50).

#### Other LILR

LILRB3 is released in a soluble from the membrane through an uncharacterized mechanism upon neutrophil activation (17). Given that soluble LILRB3 is detectable in neutrophil supernatants, binding of soluble LILRB3 to ligands could potentially possess additional functions, such as negatively regulating responses stimulated by LILRB3 ligands or by binding novel ligands not recognized by surface bound LILRB3. There is no clear evidence that other LILR are released in soluble forms from neutrophils.

## LILR Modulation of Neutrophil Response to Infection

**Figure 1:**
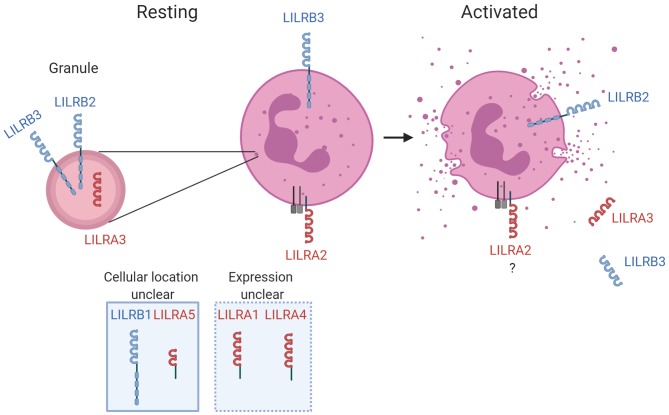
LILR in neutrophil biology. The known expression profile is illustrated for LILR on resting neutrophils and activated neutrophils. LILR detected within secretory vesicles and/or granules are shown, as well as respective up- or down-regulation from the surface during the activation process. The receptors with firm evidence of being expressed on neutrophils, or those for which further characterization is required, are illustrated for completeness. Activating and inhibitory LILR are shown in red and blue, respectively. Created with BioRender.

Given their potent immunomodulatory properties, there is growing interest in the mechanisms that regulate LILR expression. The major regulatory mechanism in neutrophils is the mobilization of SVs and granules. SVs and granules contain receptors in their membrane, and proteases and antimicrobial peptides within the luminal space. Consequently, their fusion with the cell plasma membrane during exocytosis can up-regulate surface expression of receptors they contain. Additionally, the process can down-regulate surface expression of receptors that are cleaved by the proteases released from the luminal space. Indeed, LILRB2 is upregulated through exocytosis (Figure 1). Similarly, LILRB5 is expressed in mast cell granules and upregulation is associated with exocytosis (74). LILRB3 can be rapidly released from the neutrophil surface upon degranulation (17), and proteolytic release of LILRB4 from the surface of APCs has been previously proposed (75). The up- and down-regulation of surface expression supports the notion that LILR are important modulators of neutrophil function and innate immune responses.

Emerging evidence indicates that certain LILR may have a major role in the indirect detection of infection. LILRA2 recognizes IgG and IgM that have been truncated by microbial proteases. Cleavage of antibodies into non-functional forms is an immune evasion strategy used by a broad spectrum of pathogens to reduce classical pathway opsonization, phagocytosis, and killing (76). LILRA2 interactions with N-terminally truncated antibodies stimulates neutrophils and induces antimicrobial effector functions (41). This suggests LILRA2 senses and mounts appropriate antimicrobial immune responses against a specific subset of pathogens.

There is now firm evidence that two inhibitory LILR suppress neutrophil activity. Whilst LILRB3 is downregulated upon activation (17), LILRB2 is stored within granules and is upregulated during degranulation (31). This is suggestive that LILRB3 functions to prevent premature activation, whilst LILRB2 functions to prevent overactivation. Both receptors suppress FcR-mediated neutrophil functions (17, 31). There has been no investigation of whether LILR regulate signaling of other immune receptors in neutrophils, though it is likely that LILR modulate TLR-mediated responses of neutrophils in a similar fashion to other leukocytes (32–36). However, LILR and TLR crosstalk is complicated. For example, the activating receptor LILRA2 down-regulates TLR responses (33). Thus, functional characterization of LILR-TLR crosstalk in neutrophils is required. LILRB2 and LILRB3 have been reported to detect a variety of complement split products (CSP)s including C3b, iC3b, C4b, and C4d (66). Such CSPs are produced when microbial pathogens are detected through classical, alternative or lectin activation pathways, and possess a variety of functions. As resolving complement-mediated activation is critical to maintaining homeostasis during immune responses, it is possible that interaction of CSP with LILRB2 and LILRB3 helps to prevent premature or over-reactive neutrophil activation and inflammation. Indeed, the interaction of C4d and LILRB2 was shown to suppress proinflammatory responses of monocytes (66). Additional studies need to understand whether LILR recognition of CSPs limits neutrophil activity during infection.

LILR directly recognize viral, bacterial, and parasitic pathogens. Dengue virus binds to LILRB1 but these interactions are proposed to contribute to immune evasion (53). Likewise, the human cytomegalovirus (CMV) derived UL18 protein binds LILRB1 and suppresses immune cell activity (77). *S. aureus, E. coli*, and *H. pylori* are reported to bind LILRB1- and LILRB3-expressing cell lines (36). Though the functional consequences of these interactions in human immune cells are unknown, interaction of *S. aureus* with PIR-B suppressed ERK1/2 and inflammasome activation in mouse macrophages (78). This suggests that bacterial interactions with inhibitory LILR provide immune evasion opportunities. LILRA1- and LILRB5-reporter cell lines were stimulated by *M. bovis* BCG strain (38). Functional studies are required to understand the contribution of LILR interactions to anti-*M. bovis* immune responses. *Plasmodium falciparum*, the protozoan parasite that causes malaria in humans, expresses RIFINs at the surface of infected erythrocytes that can bind LILRB1. These interactions downregulate B cell and NK cell activities against *P. falciparum* infected erythrocytes (52). Therefore, certain inhibitory LILR directly interact with microbial ligands, and these interactions provide opportunities for pathogens to suppress immune responses. Studies are now required to investigate the impact of pathogen derived inhibitory LILR ligands on neutrophil responses.

## Future Directions

Cellular and proteomic based studies have provided firm evidence that several LILR are expressed on neutrophils, and functional studies have demonstrated that LILR regulate neutrophil activation and functions. However, knowledge of the mechanisms by which LILR alter neutrophil responses in disease situations is lacking. Clarification of the LILR expression, cellular location and regulation on neutrophil is necessary, as well as better knowledge of the functional effects of LILR engagement by ligands. However, improving understanding of LILR in neutrophil biology is problematic given their short life and inability to be genetically manipulated. Further, murine PIR systems differ in number, structure and ligand interactions. The development of neutrophil-like cell lines and transgenic LILR murine lines are required to expand knowledge of LILR in neutrophil biology. Transgenic animal lines will likely be required to improve understanding of how human pathogens exploit LILR during infection.

## Author Contributions

AL and AM were responsible for drafting and editing the manuscript.

## Conflict of Interest

The authors declare that the research was conducted in the absence of any commercial or financial relationships that could be construed as a potential conflict of interest.
