# Supplementary material for: Leukocyte Immunoglobulin-Like Receptors (LILRs) on Human Neutrophils: Modulators of Infection and Immunity
Source: Front Immunol. 2020 May 13;11:857. doi: 10.3389/fimmu.2020.00857 (PMC7237751; doi:10.3389/fimmu.2020.00857)
Supplement: Supplementary file 1 [file Table_1.docx]

**Table 1: Detection of LILR-specific peptides by proteomic analysis of neutrophil-derived samples.** Black and white boxes indicate that LILR-specific peptides were detected as present or absent, respectively, in each sample. ^$^Ficloin-1 (F1) rich granules *Hypersegmented (HS) nucleus.

| **Study** | **Sample** | **LILRA1** | **LILRA2** | **LILRA3** | **LILRA4** | **LILRA5** | **LILRA6** | **LILRB1** | **LILRB2** | **LILRB3** | **LILRB4** | **LILRB5** |
| --- | --- | --- | --- | --- | --- | --- | --- | --- | --- | --- | --- | --- |
| (50) | Plasma membrane |  |  |  |  |  |  |  |  |  |  |  |
|  | Secretory vesicle |  |  |  |  |  |  |  |  |  |  |  |
|  | Azurophilic granules |  |  |  |  |  |  |  |  |  |  |  |
|  | Specific granules |  |  |  |  |  |  |  |  |  |  |  |
|  | Gelatinase granules |  |  |  |  |  |  |  |  |  |  |  |
|  | F1 rich granules^$^ |  |  |  |  |  |  |  |  |  |  |  |
| (68) | Gelatinase granules |  |  |  |  |  |  |  |  |  |  |  |
|  | Azurophilic granules |  |  |  |  |  |  |  |  |  |  |  |
|  | Specific granules |  |  |  |  |  |  |  |  |  |  |  |
| (58) | Resting neutrophil |  |  |  |  |  |  |  |  |  |  |  |
| (45) | Resting neutrophil |  |  |  |  |  |  |  |  |  |  |  |
| (43) | Resting neutrophil |  |  |  |  |  |  |  |  |  |  |  |
| (79) | Resting neutrophil |  |  |  |  |  |  |  |  |  |  |  |
| (80) | Resting neutrophil |  |  |  |  |  |  |  |  |  |  |  |
| (47) | Resting neutrophil |  |  |  |  |  |  |  |  |  |  |  |
| (46) | Resting neutrophil |  |  |  |  |  |  |  |  |  |  |  |
| (44) | Segmented nucleus |  |  |  |  |  |  |  |  |  |  |  |
|  | Banded nucleus |  |  |  |  |  |  |  |  |  |  |  |
|  | HS nucleus* |  |  |  |  |  |  |  |  |  |  |  |
| (69) | Plasma membrane |  |  |  |  |  |  |  |  |  |  |  |
|  | Secretory vesicle |  |  |  |  |  |  |  |  |  |  |  |
